# ANN-Based Intelligent Secure Routing Protocol in Vehicular Ad Hoc Networks (VANETs) Using Enhanced AODV

**DOI:** 10.3390/s24030818

**Published:** 2024-01-26

**Authors:** Mahmood ul Hassan, Amin A. Al-Awady, Abid Ali, Sifatullah, Muhammad Akram, Muhammad Munwar Iqbal, Jahangir Khan, Yahya Ali Abdelrahman Ali

**Affiliations:** 1 Department of Computer Skills, Deanship of Preparatory Year, Najran University, Najran 66241, Saudi Arabia; mahmood.mscs@gmail.com (M.u.H.); aaalawady@nu.edu.sa (A.A.A.-A.); 2 Department of Computer Science, University of Engineering and Technology, Taxila 48080, Pakistan; sifatullah.uet@gmail.com (S.); munwariq@gmail.com (M.M.I.); 3 Department of Computer Science, GANK(S) Degree College KTS, Haripur 22620, KP, Pakistan; 4 Department of Computer Science, College of Computer Science and Information Systems, Najran University, Najran 66241, Saudi Arabia; akram.moghal@gmail.com; 5 Department of Computer Science, Applied College Mohyail Asir, King Khalid University, Abha 62529, Saudi Arabia; jhkhan@kku.edu.sa; 6 Department of Information System, College of Computer Science and Information Systems, Najran University, Najran 66241, Saudi Arabia; yaali@nu.edu.sa

**Keywords:** artificial neural network, clustering, AODV, VANET, secure routing, black hole attack

## Abstract

A vehicular ad hoc network (VANET) is a sophisticated wireless communication infrastructure incorporating centralized and decentralized control mechanisms, orchestrating seamless data exchange among vehicles. This intricate communication system relies on the advanced capabilities of 5G connectivity, employing specialized topological arrangements to enhance data packet transmission. These vehicles communicate amongst themselves and establish connections with roadside units (RSUs). In the dynamic landscape of vehicular communication, disruptions, especially in scenarios involving high-speed vehicles, pose challenges. A notable concern is the emergence of black hole attacks, where a vehicle acts maliciously, obstructing the forwarding of data packets to subsequent vehicles, thereby compromising the secure dissemination of content within the VANET. We present an intelligent cluster-based routing protocol to mitigate these challenges in VANET routing. The system operates through two pivotal phases: first, utilizing an artificial neural network (ANN) model to detect malicious nodes, and second, establishing clusters via enhanced clustering algorithms with appointed cluster heads (CH) for each cluster. Subsequently, an optimal path for data transmission is predicted, aiming to minimize packet transmission delays. Our approach integrates a modified ad hoc on-demand distance vector (AODV) protocol for on-demand route discovery and optimal path selection, enhancing request and reply (RREQ and RREP) protocols. Evaluation of routing performance involves the BHT dataset, leveraging the ANN classifier to compute accuracy, precision, recall, F1 score, and loss. The NS-2.33 simulator facilitates the assessment of end-to-end delay, network throughput, and hop count during the path prediction phase. Remarkably, our methodology achieves 98.97% accuracy in detecting black hole attacks through the ANN classification model, outperforming existing techniques across various network routing parameters.

## 1. Introduction

Routing within a vehicular ad hoc network (VANET) is a complex process involving successfully transferring data packets from a source vehicle to a destination vehicle while ensuring a secure and dependable communication framework. This research primarily concentrates on achieving secure and reliable packet delivery within VANETs by implementing an innovative routing protocol using artificial neural networks (ANN). The research commences with an introduction to the background information and relevant terminologies, including a reference to a previous contribution [1]. Subsequently, an extensive review of the latest literature published in esteemed research journals is undertaken. The proposed protocol and its associated algorithms and methodology are presented in the following section. Results are presented, highlighting a state-of-the-art comparison with existing techniques, thereby underscoring the superior performance of the proposed approach in contrast to recent literature. The research concludes with a summary of findings and outlines future directions [2].

Everyday human life involves travel, with vehicles constituting an indispensable mode of transportation. VANETs form a network of nodes, primarily consisting of vehicles, interconnected through a wireless topology. Communication within this network is facilitated by RSUs [3]. Information and communication technologies (ICT) are instrumental in the intelligent transportation system (ITS), promoting safer, more efficient, thoughtful, and straightforward communication and information sharing. VANET employs wireless networks to enable efficient and remote communication among vehicles providing transportation services. However, addressing various research challenges in vehicular ad hoc networks remains formidable [4].

**Figure 1:**
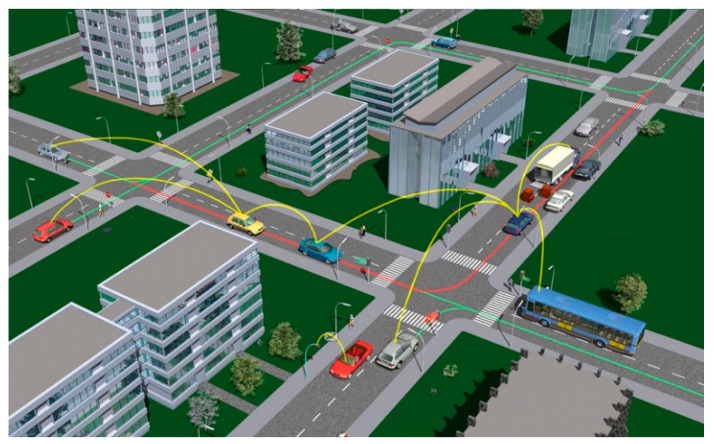
Complete vehicular ad hoc network (VANET) environment, which shows V2V, V2I, and I2I communication [13].

Firms adhering to IEEE 14712000 [5] and ISO/IEC 42010 [6] architectural standards play a crucial role in establishing VANET technology. The VANET system comprises three domains: mobile, web, and social media [7]. Within these domains, two categories, infrastructure and generic, are distinguished. The mobile domain encompasses vehicle and mobile device domains. In contrast, the infrastructure domain consists of management centers, such as traffic and vehicle management centers, and roadside devices, like traffic lights [8]. VANET systems exhibit variations based on location [9]. The CAR-2-X communication system, employed by the CAR-2-CAR communication consortium (C2C-CC) in Europe, offers a unique design and encompasses in-vehicle, ad hoc, and infrastructure domains [10]. Notably, in-vehicle communication, referred to as the in-vehicle domain, has gained increasing attention due to its implications for driver and public safety. The vehicle-to-vehicle (V2V) communication system enables the exchange of vital information and threat notifications among vehicles, enhancing driver assistance. The vehicle-to-road infrastructure (V2I) communication system provides access to real-time traffic, weather, and environmental data [11]. Figure 1 illustrates VANET communications. The utilization of wireless broadband techniques, such as 3G/4G, in vehicle-to-broadband cloud (V2B) communication systems offers substantial benefits, enabling communication between vehicles, active driver assistance, and vehicle tracking, thanks to the increased data storage capacity provided by broadband cloud [12].

These data can be in different forms, such as real-time weather updates and traffic information, infotainment, and environment data. Figure 1 shows the communication between vehicles [14].

### 1.1. Navigating Vehicular Ad Hoc Networks

When crafting routing protocols for VANETs, it is imperative to consider the trifecta of security, mobility, and scalability. These routing protocols delineate the mechanisms by which two nodes or routers communicate within a computer network [15]. Additionally, these protocols are instrumental in discerning the optimal candidates to steer data packets along their intended path. It is worth noting that many factors, including road conditions, traffic dynamics, physical obstacles such as buildings, and the broader environmental context, significantly influence the routing protocols in VANETs. Additionally, the dynamics of vehicular movement and potential disruptions within the network further contribute to the complexity of these protocols. The ad hoc routing protocols, crucial in VANETs, can be neatly categorized into three distinct types: hybrid, proactive, and reactive, each offering unique approaches to tackling the intricate challenges of vehicular communication [16].

### 1.2. Clustering Strategies in Vehicular Ad Hoc Networks

Within the VANETs framework, clustering assumes pivotal importance, facilitating the assembly of a group of vehicles with similar attributes. In the realm of VANET clustering, the mobility-based approach stands as the most prevalent method. However, several clustering techniques in VANETs have harnessed sophisticated algorithms such as fuzzy logic and machine learning to enhance the stability and efficiency of these clusters [17]. VANET clustering deploys a multi-hop-oriented approach and leverages network mobility (NEMO) techniques. Depending on the specific algorithms in use, the clustering technique in VANETs may opt for either the single-hop or multi-hop approach. Single-hop techniques, in turn, can be further categorized into intelligence-driven and mobility-centric methods [18].

**Figure 2:**
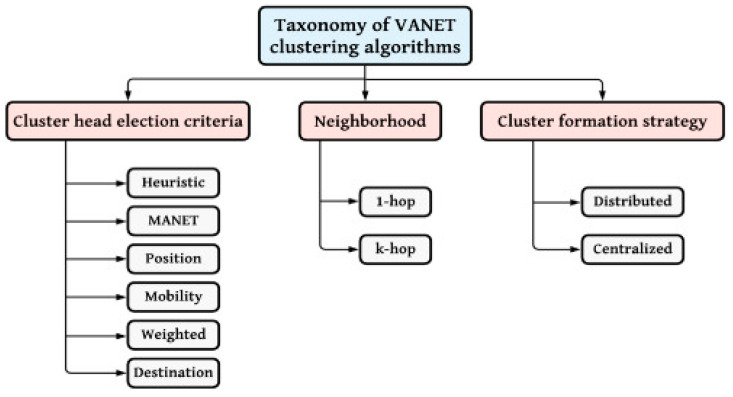
VANET clustering strategies for cluster head selection, neighbor nodes selection, and cluster formation strategy [19].

Furthermore, VANET clustering is enhanced by integrating fuzzy logic into the fray. In the comprehensive VANET approach, the fuzzy logic algorithm is artfully amalgamated with supervised learning algorithms, including Q-learning and machine-learning clustering techniques. This union develops a versatile and efficient clustering mechanism within the context of VANETs. Figure 2 shows the taxonomy of VANET clustering algorithms, which includes all the clustering algorithms’ techniques.

The preeminent clustering methodology employed within VANETs is the mobility-oriented clustering technique. This technique pivots around essential metrics rooted in the dynamics of vehicular movement, encompassing parameters such as acceleration, relative speed, positional data, and the direction of vehicular motion, among others. A key challenge in the VANET context is the imperative need to optimize the cluster count. Several clustering techniques predicated on multi-hop packet transmission have been documented in the scientific literature to address this concern, all aimed at curtailing the number of clusters. This approach is pivotal as it allows a cluster head (CH) to extend its coverage over a more expansive geographical area, engendering heightened network stability [20].

Within the domain of multi-hop strategies, two distinct categories emerge. In the context of two-hop communication, the cluster head is empowered to oversee vehicular communication spanning up to two hops, encompassing a segment of vehicles within its purview. The formation of clusters hinges upon a routing technique that considers vehicle positions and their respective traveling directions. Conversely, in the ambit of 2+ hop algorithms, the cluster head can expand its reach across two or more hops, a scenario witnessed in various multi-hop-based algorithms, such as those catering to three-hop, four-hop, or even five-hop coverage scenarios.

### 1.3. Cyber Attack Scenarios in VANET

**Figure 3:**
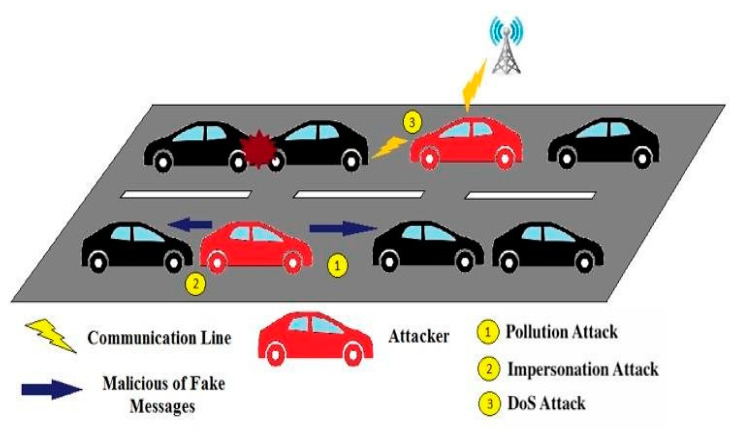
Illusion of pollution, impersonation, and DoS cyber attack scenarios in VANET [26].

Security in VANETs is profoundly influenced by a spectrum of attacks that can significantly impede the performance of various routing protocols [21,22,23]. These attacks encompass diverse categories and are pivotal to elucidating fundamental forms of routing attacks, as delineated in Figure 3. In a compromised vehicle scenario, malicious nodes exploit vulnerabilities by initiating pollution, impersonation, and denial of service (DoS) attacks. The compromised vehicle emits fake messages to nearby vehicles, introducing pollution attacks that manipulate environmental data. Impersonation attacks involve the malicious node posing as a trusted entity, deceiving neighbouring vehicles with falsified information.

Additionally, the compromised vehicle executes DoS attacks, overwhelming the communication channels of nearby nodes to disrupt their normal functioning. These orchestrated attacks compromise the integrity of the vehicular network, jeopardizing communication, trust, and overall system reliability. Effective cybersecurity measures are crucial to mitigate such threats and ensure the secure operation of connected vehicles. First, we categorize these attacks into two primary forms: active and passive. In an active attack, the malevolent actor intercepts network data and manipulates the message’s content. This results in the intended recipient receiving a message that has been tampered with. The motives behind active attacks are often centered around undermining network efficiency or exploiting potential vulnerabilities to gain unauthorized access to provide illicit services. This transpires through eavesdropping on wireless networks, data collection, or the probing of possible network weak points. On the contrary, passive attacks manifest when an assailant abstains from sending or receiving network messages directly. The attacker’s role is observatory and data collection rather than active interference [24].

Conversely, the rational attacker attacks to gather intelligence and harm the network. The attacks are further distinguished by their scope, with local and extended attacks forming a dichotomy. Local attackers initiate assaults confined to a limited geographical area, typically affecting specific RSUs and nodes. In contrast, extended attackers extend their impact over a broader expanse, intending to degrade network efficiency or render it completely non-functional. In the context of this research, the focus is on routing attacks in VANETs. There are precisely five distinctive types of routing attacks: a denial of service (DoS) attack overwhelms a system, making it unavailable to users by flooding it with excessive traffic, disrupting standard functionality. A black hole attack shows malicious nodes in a network drop or absorbs incoming data, rendering the affected paths unusable and disrupting communication. A gray hole attack is an attack in which a compromised node selectively drops or modifies data packets, causing inconsistencies in communication while maintaining some level of standard functionality. An illusion attack fakes the presence of non-existent nodes or services, deceiving neighboring nodes regarding the network topology and leading to misrouting of data. A sinkhole attack occurs when network traffic is diverted to a malicious node, allowing the attacker to intercept and manipulate the data flow and compromise communication integrity [25]. These attacks pose significant challenges to the integrity and security of data transmission within VANETs.

### 1.4. Machine Learning in VANET Routing Prediction

In the realm of vehicular ad hoc networks (VANETs), the integration of machine learning techniques for routing prediction has emerged as a pivotal area of research. One noteworthy approach involves utilizing supervised learning models such as support vector machines (SVM) and random forests. SVMs excel in classifying and predicting data by finding the optimal hyperplane that separates different classes, making them well-suited for discerning intricate patterns in vehicular movement. On the other hand, random forests leverage the collective decision-making power of multiple decision trees, enhancing the robustness of predictions. Their ability to handle large datasets and adapt to dynamic network conditions positions them as formidable tools in predicting optimal routes within VANETs. These models offer advantages over traditional routing algorithms by discerning patterns in real-time traffic data, considering factors like congestion, road conditions, and vehicular movement, resulting in more adaptive and efficient routing decisions.

**Figure 4:**
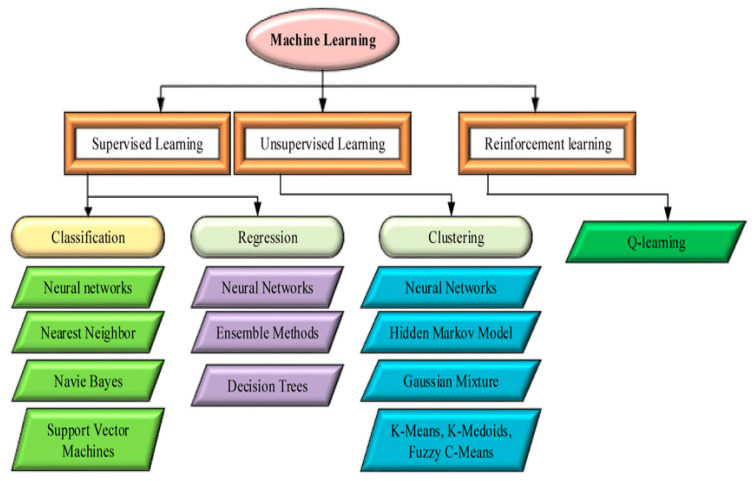
Machine learning models used in VANET routing prediction [27].

Furthermore, the advantages of machine learning-based routing prediction extend beyond individual model choices. Figure 4 shows Machine Learning models used in VANET routing prediction. Ensemble methods, which combine the strengths of multiple models, have demonstrated exceptional performance in enhancing prediction accuracy and generalization. Hybrid models that integrate machine learning with traditional routing algorithms capitalize on the strengths of both approaches, offering a balanced solution that benefits from the adaptability of machine learning and the stability of conventional routing methods. The dynamic nature of vehicular environments necessitates models that can adapt to changing conditions, and machine learning techniques in VANET routing prediction pave the way for intelligent, context-aware decision-making, ultimately contributing to safer and more efficient transportation systems. 

### 1.5. Research Motivation

Efficiency and security in routing are paramount for enhancing network performance. Ensuring the availability of optimal pathways in a vehicular network, preserving privacy, and safeguarding data integrity are central concerns that necessitate focusing on security matters. The operational effectiveness of VANETs is notably impacted by various security attacks, exacerbated by the absence of a precise defence mechanism. The lack of a centralized monitoring system, the implementation of cooperative algorithms, the dynamic nature of network topology, and the limited transmission range of nodes collectively give rise to an array of routing challenges. Consequently, a pressing need arises for secure and intelligent routing techniques to minimize communication delays and identify security threats.

The primary objectives of our research are as follows:

- The development of a cluster-based routing technique to facilitate the efficient delivery of content.
- The detection of malicious nodes in VANET through the application of deep learning.
- The establishment of optimal pathways to ensure the efficient delivery of data to their intended destinations.

A principal concern in the realm of VANET data transmission pertains to secure and efficient routing. This is a non-trivial task, primarily due to the mobility of nodes within a network characterized by both homogeneity in vehicular nodes and heterogeneity in VANET contexts. Routing efficiency is frequently compromised by packet transmission delays caused by unsecured or malicious routes, often involving multiple intermediary nodes. Thus, a compelling imperative arises for developing an effective and intelligent technology that can enhance routing efficiency by minimizing the hop count while bolstering security with minimal data loss. Consequently, our proposed technique combines secure and efficient packet routing, substantially reducing communication delays. A cluster-based secure routing approach minimizes the hops required for content delivery, while a machine learning-based method is harnessed to ensure security. This innovative approach is tailored to enhance routing efficiency and reliability. The primary objective of our proposed model is to establish a secure cluster-based routing framework within VANETs specifically designed to address secure content requests and consumption. Routing security is upheld by integrating machine learning techniques and on-demand routing, implemented through a modified AODV protocol.

### 1.6. Problem Statement

**Figure 5:**
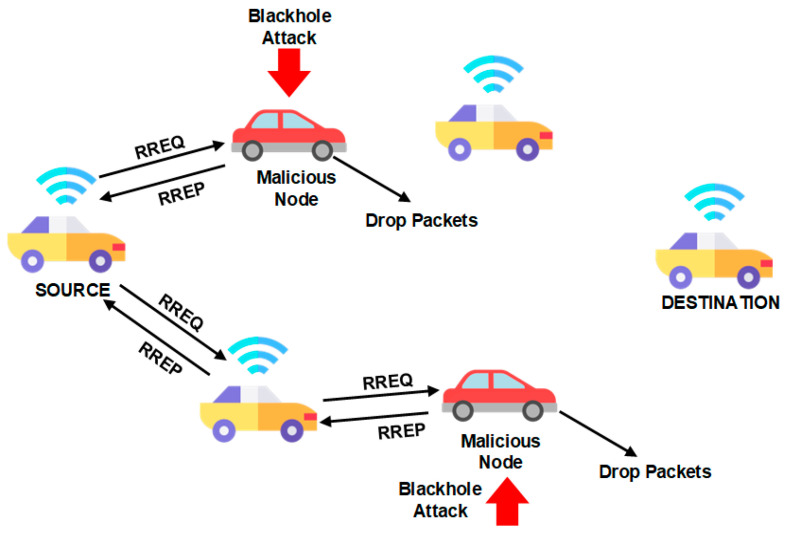
Problem diagram under black hole attack scenario in network for packet loss.

**Figure 6:**
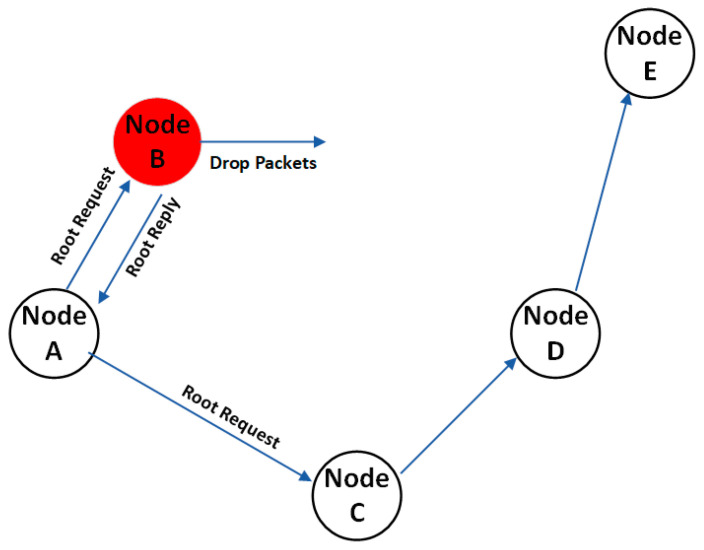
Routing attack scenario of proposed approach.

Ensuring secure and efficient data transmission in vehicular ad hoc networks (VANETs) is crucial due to dynamic nodes in a mix of homogenous and heterogeneous VANET environments. Packet delays, insecure routes, and intermediary nodes hinder routing efficiency. Mobility patterns and hop count challenges cause delays in data exchange for content sharing. VANETs face attacks like black holes, flooding, and DDoS. In black hole attacks, malicious nodes divert packets from their path, leading to a “black hole” effect where packets are discarded. Figure 5 illustrates this issue, impacting communication and causing breakdowns. To address this, we propose a machine learning-based, secure, and efficient routing approach to mitigate malicious node attacks and reduce packet transmission delays, as shown in Figure 6.

### 1.7. Research Contributions

The principal research contributions of this proposed work are outlined as follows:

- An innovative, secure routing protocol was introduced to combat black hole attacks within VANETs. This protocol is designed to optimize the allocation of tasks by selecting the most secure and efficient path for data transmission.
- Integrating a cluster-based routing technique facilitates the streamlined delivery of content to the target vehicle following a meticulous verification process based on the vehicle’s past performance, utilizing ANNs.
- Establishing clusters through a meticulously designed algorithm incorporating security considerations further bolsters the dependability of the hop count in the network.
- The deployment of ANN techniques to identify and combat black hole attacks and secure routing within VANETs. This approach leverages knowledge and related skills to pinpoint routing paths between source and destination nodes accurately.
- Implementing an authentication-based method for efficient content delivery ensures secure analysis and path prediction. This is achieved by utilizing a modified AODV algorithm, which effectively predicts the route after securing the network using an ANN-based machine learning (ML) algorithm.

Thorough performance analysis of the proposed technique, featuring a comparative evaluation against AODV-L, AODV-R, and T-AODV. This assessment encompasses critical metrics such as end-to-end delay, average hop count, network throughput, and other relevant performance indicators expounded upon in this paper.

The subsequent sections of this paper adhere to the following organizational structure.

- Section 2 explores related work focused on secure routing within VANETs, shedding light on previously adopted methodologies. Various established secure routing solutions are presented alongside examining multiple clustering techniques. Towards the conclusion of this section, diverse machine-learning methods for detecting malicious nodes are scrutinized.
- Section 3 intricately expounds upon the proposed solution framework and model diagram. Secure routing is realized through a cluster-based machine learning model and a modified AODV algorithm. This section elucidates the proposed solution by incorporating diverse flowcharts and model diagrams, ensuring a lucid comprehension of the methodology.
- Section 4 involves simulating our techniques within a suitable simulation environment, utilizing the NS-2 simulator and Python for machine learning. Existing datasets are harnessed for model training, and this section offers a comprehensive description of the experimental setup and various components of the experiments.
- Lastly, Section 5 concludes the paper, and potential avenues for future research are delineated.

## 2. Literature Review

Various methodologies within existing routing and security approaches have been extensively explored in the available literature. In conjunction with the enhanced dragonfly algorithm, the k-medoid clustering model has emerged as a notable contributor to improving energy efficiency within vehicle-to-vehicle communication [20]. The proposed approach entails the selection of medoids through the calculation of distances between data points. Initially, each object is assigned the nearest medoid value, which is subsequently refined to its closest medoid value. The cluster head selection depends on carefully assessing speed, position, and acceleration parameters. By harnessing the k-medoid clustering model alongside the enhanced dragonfly algorithm, the transmission of messages to various vehicles, RSUs, and base stations is efficiently minimized, culminating in substantial energy savings within the vehicular network. This combined approach also contributes to a heightened packet delivery ratio and network throughput, albeit without a specific focus on the hop count metric.

Furthermore, clustering has been a favored strategy among many researchers for facilitating reliable and secure routing. A credible clustering technique aimed at optimizing communication was proposed by [28]. In this instance, the parameters of velocity and distance are crucial considerations for forming clusters. The determination of a distrust value plays a pivotal role in selecting a trustworthy cluster head. Should the distrust value exceed a predefined threshold, the vehicle in question is added to a blocklist, whereas a distrust value below or equal to the threshold results in the vehicle being added to an allowlist. Emphasis is placed on considering a vehicle with higher trust as the cluster head. Following the selection of a trustworthy cluster head (CH), the clustering quality of service (QOS) value is meticulously computed. This clustering methodology significantly enhances the packet delivery ratio and throughput, concurrently mitigating packet loss and end-to-end delay issues.

### 2.1. Effectual Routing Strategies

A novel approach grounded in fuzzy logic is posited to enhance routing efficiency within VANETs [29]. This study endeavours to augment the AODV protocol by infusing fuzzy logic rules. The construction of a routing table underpins the proposed technique, and these fuzzy rules determine the route selection with the lowest hop count. A threshold value is introduced to govern the propagation of routing packets within the vehicular network. Each node’s choice of an appropriate neighbor for packet transmission is influenced by specific parameters such as velocity, direction, and distance. The threshold value’s calibration is contingent upon the network’s density. When a source node dispatches a route request to neighboring nodes, each recipient node scrutinizes the request packet, calculating the link expiration metric and the link reliability model. These calculations, constituting inputs to the fuzzy system, are based on parameters like velocity, direction, and distance. At the receiving end, notably the destination node, fuzzy inputs are harnessed to compute the fuzzy cost for each prospective route, culminating in selecting the route boasting the highest cost function. Various routing protocols are at the forefront of efficient packet delivery. Within the proposed approach [30], routing decisions are executed at intersections with the assistance of roadside units. The process commences with the transmission of a beacon message by the RSU, aimed at discovering and sustaining a link between itself and neighboring nodes, facilitated by a link discovery beacon. After link establishment, real-time information about lane availability, the direction of the next-hop intersection, and traffic flow is harnessed to ascertain a triangular fuzzy number. Per our proposed method, packets with the highest fuzzy score are dispatched to the intersection. Utilizing a greedy forwarding approach augments the link lifetime or strength between two intersections, a parameter contingent upon temporal and nodal velocity considerations. This optimization contributes to a reduction in end-to-end delay, packet routing delay, and hop count.

In our proposed approach [4], a secure routing algorithm is devised to detect and thwart black hole attacks. The AODV routing method is employed with tailored modifications for detecting malicious nodes and facilitating efficient packet delivery. Cryptographic encryption and decryption techniques serve to verify the identities of both the source and destination nodes. Within our proposed approach, the source node conducts an initial assessment of the status of neighbouring nodes before generating an encrypted packet. Subsequently, the encrypted packet is transmitted to these neighboring nodes, and their presence is ascertained within the loop. The requisite secret key is transmitted to the identified neighbor node. Following the scrutiny of packet request and response data, the packet is relayed to its ultimate destination; the secure routing protocol known as TAD-HOC routing protocol was introduced in [31]. AODV is coupled with group authentication keys to ensure secure packet delivery with minimal delays. Our methodology hinges on the sequential reception of TROPHY messages by authenticated nodes. Four key entities play integral roles in creating a secure environment: onboard units, RSUs, key distribution centers (KDC), and human operators. The KDC generates periodic keys, subsequently disseminated to nodes for authentication. If a node is not actively participating in the routing process, KDC and RSUs swiftly initiate refreshing and updating the routing paradigm by expunging superfluous or malicious nodes and disseminating information regarding the identified malicious nodes to all other network nodes. In the event of a node’s abrupt disconnection from the network, KDC initiates data recovery procedures and essential refreshment processes. It facilitates the retrieval of data about all inactive nodes, ultimately leading to their permanent removal from the routing process.

Ref. [32] introduced a modified TAD-HOC routing mechanism featuring a discerning forwarding strategy. In this approach, the utilization of AODV is complemented by group authentication keys to ensure the secure and expeditious delivery of packets. In the framework we propose, TROPHY messages are meticulously received in sequence exclusively by nodes that have been duly authenticated. Four distinct entities synergize within our proposed approach to establish a fortified network environment: onboard units, roadside units, key distribution centers (KDC), and human operators. The KDC serves as the keystone, generating cryptographic keys regularly and disseminating them to network nodes for robust authentication. If a node is not actively engaged in the routing process, the KDC and RSUs proactively refresh and renew the routing infrastructure. This entails meticulously removing redundant or malevolent nodes and disseminating pertinent information regarding the identified malicious nodes to all other network constituents. A judicious forwarding algorithm is harnessed to detect extra packets, and once redundant packets are identified, the messages are transmitted in a covetous fashion. Our protocol exhibits a remarkable 92% efficiency gain in comparison to TAD-HOC. The authors in [33] discuss a system for trust-based secure routing in vehicular ad hoc networks (VANETs) using deep learning-based attack detection. Here is an analysis of the reference’s advantages and disadvantages compared to your work and a hypothetical comparison of results. The first advantage is the hybrid approach that combines an ANN model for attack detection with a modified AODV protocol for routing. This combination can provide a comprehensive solution for VANET security and routing. Detection of black hole attacks addresses the problem of black hole attacks, a significant security concern in VANETs. Using an ANN model, the system claims to achieve a high accuracy of 98.97% in detecting black hole attacks, a crucial advantage in securing data transmission. Cluster-based routing employs cluster-based routing, enhancing network organization and reducing routing complexity in VANETs. Performance evaluation mentions a thorough evaluation of the system’s performance using various metrics such as accuracy, precision, recall, F1 score, and loss. It also considers network parameters like end-to-end delay, network throughput, and hop count, comprehensively assessing routing efficiency. It outperforms existing techniques, demonstrating its potential as an improved VANET security and routing solution. Disadvantages include lack of implementation details: the reference does not provide detailed information on the implementation of the proposed system, making it challenging to replicate the work or assess its real-world feasibility. Limited information on the ANN model: while the reference mentions the use of an ANN model for attack detection, it lacks information about the architecture and training process of the model. This omission can be a drawback for those interested in replicating or improving upon this aspect of the work. Evaluation using a specific dataset states that it uses the “BHT dataset” for evaluation, but it does not provide details about this dataset. The suitability and representativeness of the dataset can be necessary for the credibility of the results.

To compare your work with the proposed system, compare the methodologies of your work and the reference. Assess whether the hybrid approach and ANN-based attack detection are similar or different from your approach. Results show that if you have conducted experiments and evaluations in your work, compare your results with those presented in the reference. Look for differences in performance metrics, such as accuracy, precision, recall, F1 score, and routing efficiency. Implementation evaluates the practical implementation aspects of your work compared to the reference. Consider the ease of implementing your solution in a natural VANET environment. The proposed research compares the dataset to the “BHT dataset” mentioned in the reference. Assess the relevance and representativeness of the datasets used in both works.

Similarly, ref. [34] introduced an efficacious routing algorithm known as the Dyte algorithm, designed for optimizing the delivery of packets while concurrently minimizing packet delays. Within the framework of the Dyte algorithm, a trilateral zone is thoughtfully delineated by the source node, precisely ascertaining the final location of the destination node. This delineation culminates in compiling a roster of nodes encompassing the last known location of the destination node. The source node appends the destination node’s identity and other pertinent information to the packet header before dispatching it to the node located within the trilateral zone. Every constituent node within the trilateral zone scrutinizes the packet’s information and responds as dictated by its operational state. Should a node possess knowledge of the destination node’s whereabouts, an additional trilateral zone is meticulously charted. Subsequently, a novel packet is engendered and directed towards the destination. Conversely, in cases without the knowledge of the destination, intelligence regarding the trilateral zone is shared with neighboring nodes until the message reaches the intended destination.

### 2.2. Machine Learning-Based Approaches for the Identification of Malicious Nodes

Various machine-learning techniques have been applied to identify malicious nodes within the existing body of research. The work presented by [27] introduces a trust-based machine learning approach employing the k-nearest neighbors (KNN) algorithm and fuzzy logic for clustering. Trust is ascertained using the beta distribution method based on three fundamental values: trust, reputation, and reputation dependency. Each node’s reputation is intricately linked to its level of trustworthiness. Following the trust evaluation, the derived trust values are transmitted to the cluster head, which subsequently updates the reputation values of individual nodes. Upon completing the clustering and trust assessment, the presence of malicious nodes is discerned. This is achieved by having the source node dispatch a route request to the cluster head, scrutinizing the destination node’s presence within the cluster. If the destination node is not located within the cluster, the cluster head initiates communication with the corresponding cluster head in other clusters. The evaluation of trust and reputation for each node is meticulously conducted, and any indications of malevolent activities prompt the blocklisting of the respective node. In a distinct study by [35], machine learning techniques and statistical models are leveraged to identify spurious nodes. The proposed methodology evaluates the efficacy of various classifiers in pursuing malicious node detection. These classifiers include the gradient boosting classifier, support vector machine, k-nearest neighbors classifier, logistic regression, and naive Bayes classifier. From the pool of 19 extracted features, the top 7 features are selected for evaluation, encompassing parameters like throughput, lost packets, source node IP address, source and destination port, time of the first packet received, and time of the last packet received. The routing protocol adopted is AODV, and the NS-3 simulator is utilized for the experimental framework. The gradient boosting classifier emerges as the most effective choice for malicious node detection.

Ref. [36] introduces a misbehavior detection technique founded upon a machine learning algorithm. This approach unfolds through a sequence of four key steps. The first phase involves data acquisition by collecting information from sensors such as GPS and kinematics. Subsequently, various features are extracted from the acquired data. In the second phase, data on neighboring nodes is gathered, and specific characteristics of counterfeit nodes are isolated. These attributes include node behavior, packet communication delay, and broadcasting ratio. The third phase focuses on feature derivation and model training using supervised learning techniques. Once the model is suitably trained, malicious nodes are identified by applying an ANN classifier.

Ref. [37] introduced a machine-learning approach to detect malicious nodes within the mobile ad hoc networks (MANETs) context. This innovative approach couples the AODV protocol with ANN and support vector machine (SVM) techniques to bolster routing security. SVM is leveraged to identify abnormal behavior exhibited by nodes encountered along the path established by AODV. Upon singling out a suspicious node, ANN is deployed to assess the accuracy of SVM outcomes, thereby ascertaining whether the flagged node is fraudulent. The existing body of literature delves into many techniques to enhance the efficiency and security of routing. VANET clustering techniques are employed diversely to optimize routing efficiency and prolong network lifespan. The amalgamation of fuzzy logic with AODV and various AODV routing strategies is explored to make informed routing decisions. In parallel, AODV is harmonized with distinct machine learning methodologies and conventional practices to identify and combat black hole attacks in vehicle-to-vehicle (V2V) communication scenarios. Typically, the evaluation of routing efficiency hinges upon key metrics such as packet delivery ratio, end-to-end delay, hop count, and packet drop ratio. However, a sophisticated, security-oriented infrastructure-based routing approach focusing on robust network throughput remains absent. Consequently, our proposed method offers a productive and secure routing paradigm for VANETs. By fusing a fitness function and integrating forward and backward control packets for short path prediction alongside a supervised deep learning model for malicious node detection, this approach significantly enhances routing efficiency and reliability regarding average end-to-end delay, hop count, and network throughput [38].

Despite the prominence of VANET research, particularly in data delivery and routing, the field remains relatively underexplored regarding security. While the literature encompasses many techniques, including AODV protocols, TAD-HOC routing protocols, cluster-based routing, Dyte algorithms for optimizing packet delivery efficiency, fuzzy logic-based routing decisions, and similar approaches, these predominantly focus on routing solutions. These approaches often lack a comprehensive consideration of potential attacks on the vehicles involved in the routing process, instead concentrating on statistical and mathematical results [39]. Moreover, they overlook the integration of machine learning (ML) techniques, such as ANNs. In light of these limitations, we seek to address the gaps that persist within the existing approaches. Our results endeavor to present the ANN-based intelligent secure routing protocol for vehicular ad hoc networks. This protocol delivers substantial enhancements across various facets of network performance, particularly in detecting black hole attacks, end-to-end delay for packet transmission, transmission efficiency, hop count, and network throughput. The findings presented in the concluding section, Section 5, underscore the comprehensive efficiency and enhancements of our proposed network solution.

## 3. Intelligent Cluster-Based Routing Approach

In vehicular ad hoc networks (VANETs), the intricate interplay of content placement and delivery necessitates nuanced consideration, primarily attributed to the dynamic mobility characteristic inherent to the VANET environment. Crafting a secure routing protocol for content placement and delivery is paramount in navigating this scenario. The foundational step lies in the thoughtful design of the VANET network, specifically concerning the exchange of vital information. Within the ad hoc network paradigm, three critical modes of communication come to the fore: route error (RRER), route request (RREQ), and route reply (RREP). The innovation introduced here rests upon a secure, cluster-based routing mechanism that strives for efficiency and security. Our proposed routing protocol is structured to provide precise information that promotes efficient content delivery and related placement strategies. Leveraging the mobility dynamics inherent in the system, we present an efficient cluster-based secure routing protocol meticulously tailored to minimize content request delays.

### 3.1. Routing Attack Scenario

In the context of VANET, communication delays are an inevitable consequence of its open and peer-to-peer communication structure. Within our proposed methodology, these delays are primarily attributed to the robust and influential group-based management policy that underscores the system. A salient concern stems from the number of intermediary nodes that come into play when two communication vehicles interact within VANET. The openness of this environment brings to light two critical issues related to black hole node attacks. Firstly, there is the matter of path availability, and secondly, the identification of malicious nodes. Notably, not every vehicle can relay a path to other nodes. Against this backdrop, the proposed routing approach contemplates and addresses black hole attacks. These nefarious nodes initiate their assault by targeting nearby nodes with a carefully orchestrated scenario, aiming to disrupt the communication chain from the outset. At the inception of its operation, the attacker node transmits the shortest route to another node. Subsequently, upon reaching the midpoint along the shortest path, it subtly withholds packet transmission, thus diverting packets away from the intended path. This surreptitious packet withholding engenders the characteristic black hole effect, resulting in the drop of all packets traversing that route, rendering them incapable of reaching their intended destination. The attack scenario and its ramifications, specifically concerning the mitigation of distributed denial of service (DDoS) attacks, are elucidated in Figure 6.

In addition to the concern, the intricacies of routing in VANET consistently pose a formidable challenge owing to network nodes’ rapid and dynamic movement. These VANET nodes encompass both homogenous and heterogeneous elements, with the vehicle-to-vehicle (V2V) environment significantly influencing content behavior. Transmitting or receiving data for content sharing and placement is impeded by the transient nature of node mobility and the resultant delays in the hop count within the VANET infrastructure. To tackle this issue, we introduced an intelligent cluster-based secure routing protocol to mitigate content request delays.

### 3.2. The Optimal and Secure Route Minimizing Communication Latency to the Fullest Extent

This paper introduces a resolution that articulates an ideal and secure pathway, minimizing the communication delay within the designated vehicle network. $V_{N}$ is the vehicular node, and N = { $N_{1},N_{2},\ldots .N_{m},$ } is the number of vehicles. Data are sent from the ${VN}_{s}$ source node to the ${VN}_{d}$ . We collect the information through all the nodes between the source and destination nodes using $\sum_{i=0}^{n}{Vn}^{i}.$ We set the location of the vehicles using $V_{cl}^{Loc[x,y]}$ under the velocity of nodes using $V_{cl}^{vel}$ . There are several nodes on the maps that provide the directions from one node towards others, so we use ${map}_{lane}$ under timestamps to provide the node’s location through any distance from RSU. The threshold distance is set using $D_{threshold}$ under $D_{RSU}$ nodes selections.

### 3.3. Intelligent Path Prediction

**Figure 7:**
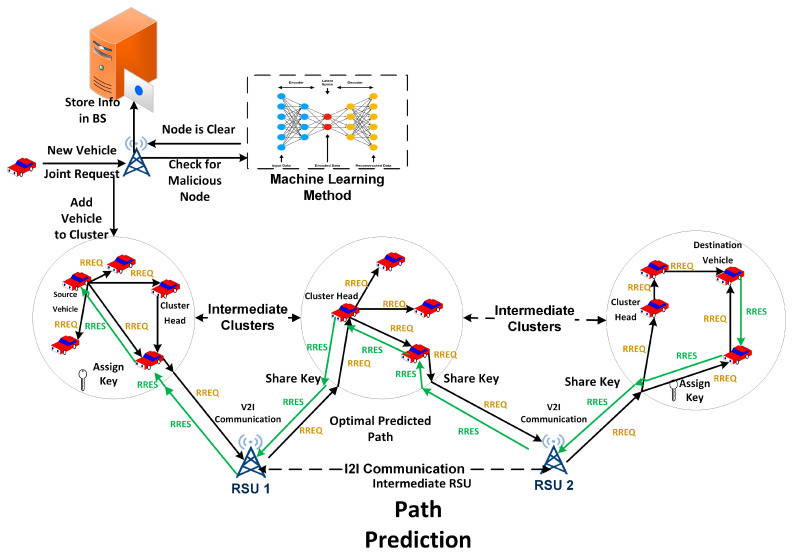
Methodology of the proposed system.

Our devised method operates under the assumption of a uniform dispersion of vehicles across the urban landscape, forming two-dimensional links. The transmission, denoted as Tr, is distributed uniformly throughout the vehicular ad hoc network (VANET) environment. Vehicle “p” is regarded as the neighboring node of vehicle “q”, and the transmissions between these vehicular nodes (V-Nodes) should not exceed a specific range denoted as “r”. Figure 7 illustrates the methodology of the proposed system.

We set the BS as the base station to collect vehicle information and apply ANN-based implementation under a modified AODV algorithm concerning trustworthy nodes. $T_{R}^{N}$ machine learning makes these trust nodes $T_{R}^{ML}$ . The vehicle requests a route. $V_{R}^{root}$ for data-sending and then receiving replies through $V_{R}^{root}$ .

**Figure 8:**
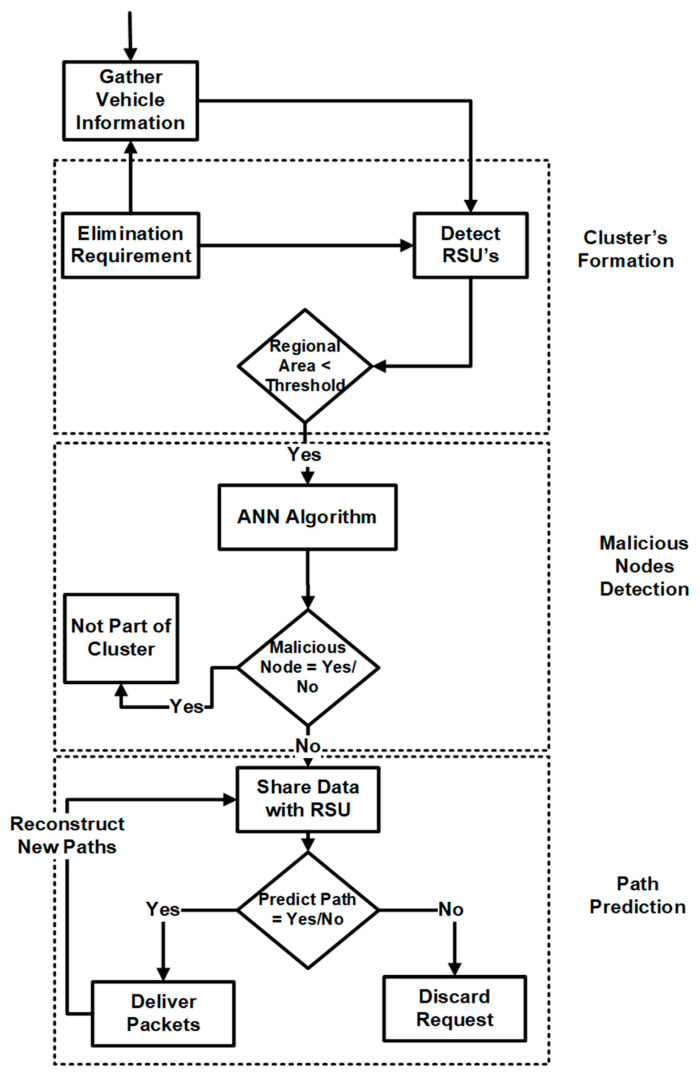
Flow diagram for optimal path prediction and packet transformation of the proposed approach.

A uniform speed profile is maintained across all vehicles within our framework. We delineate two primary lanes connecting the source and destination nodes. Each vehicular node (V-node) employs a singular algorithm, affording a solitary opportunity to succeed within the VANET environment. This algorithm facilitates intercommunication between different waypoints. Thanks to our method, minimal interference with the driving behavior of vehicle nodes results in substantial control over other network participants. A routing protocol is employed to adhere to prescribed routing instructions issued by the RSU, as shown in Table 1. Failure to comply with RSU instructions for request or response routing results in non-disclosure of routing and other vital information, ensuring its security. VANET proves instrumental in efficiently delivering and placing content to serve vehicular nodes. VANET augments content request and response parameters by incorporating effective monitoring and control measures. Our proposed methodology is visually represented in Figure 8, encompassing vital components such as malicious node detection through machine learning, cluster formation, and efficient path prediction utilizing a modified AODV protocol. In the initial phase, nodes undergo evaluation by the RSU through a deep learning model. Upon satisfying the requisite clustering parameters, nodes are integrated into clusters, and optimized communication paths are predicted using the modified AODV protocol. This process is elucidated in Figure 8. Modified AODVs (ad hoc on-demand distance vector) enhance the traditional AODV routing protocol used in mobile ad hoc networks (MANETs). The modifications in this protocol aim to improve the routing efficiency, reduce latency, and enhance its adaptability to the dynamic nature of MANETs. The main reasons for the modification of AODVs are as follows:

- Enhanced route discovery: Modified AODVs employ advanced algorithms to optimize the route discovery process. It uses techniques like improved route request broadcasting and route reply propagation. This enhancement reduces the time taken to establish a route.
- Classification parameter: The modifications introduce a classification parameter that helps categorize routes based on their stability and reliability. This parameter is used to select the most suitable route for data transmission.
- Dynamic route maintenance: Modified AODVs adapt to network changes more efficiently. It incorporates mechanisms for monitoring the stability of routes and triggers route updates when necessary. This ensures that the protocol can quickly react to link failures and changes in network topology.
- Load balancing: The modified version is designed to distribute traffic evenly across available routes, preventing congestion and improving overall network performance. This is achieved through route selection algorithms that consider the current network load.
- Improved scalability: The modifications address scalability concerns by optimizing the route table management. It can handle a larger number of nodes in the network without a significant increase in control overhead.
- The main advantage of this AODV modification is that it enhances the proposed system’s performance. Reduced latency: The improved route discovery and maintenance mechanisms reduce the latency in establishing and maintaining routes. This is crucial for real-time applications like video streaming and online gaming.
- Enhanced reliability: The classification parameter helps select more reliable and stable routes, reducing packet losses and improving communication quality.
- Adaptability: The protocol can quickly adapt to changes in the network, such as node mobility or link failures, making it suitable for dynamic ad hoc networks.
- Efficient Resource Utilization: Load balancing and scalability improvements ensure that network resources are utilized efficiently, leading to better network performance and longevity.
- Improved quality of service support: The modifications enhance the support for quality of service (QoS) by allowing for a better selection of routes based on specific application requirements.

### 3.4. Cluster Formation

In this approach, the roadside unit (RSU) is pivotal in guiding the formation of clusters, as detailed in Algorithm 1. Initially, each vehicle node enters the simulation environment, represented as a lane on the highway, and broadcasts a timestamp. Cluster’s C are transformed after setting, causing range through $C_{i}^{Range}$ . After setting the cluster with range, each cluster is assigned an identity using $C_{i}^{identity}$ . In each cluster, the number of neighbors is setup through $C_{neighbour}$ .

These vehicles initially establish communication with all RSUs within their operational range. To achieve robust and stable cluster formation, we employ four critical conditions. These conditions consider the relative change in speed (denoted as $V_{cl}^{val}$ ), the distance (D) covered by the vehicle during the timestamp, and the number of neighboring vehicles within the range of cluster nodes. The distance between an RSU and a new vehicular node is computed upon entering the system. According to Algorithm 1, the vehicular node is affiliated with the RSU if this distance is below a certain threshold. The RSU plays a central role in cluster formation, managing content distribution, message requests, and responses. Initially, vehicles broadcast timestamps to RSUs, initiating the formation of clusters using Algorithm 1. The RSU oversees the establishment of connections, employing conditions based on speed, distance, and neighboring vehicles. The RSU monitors vehicular nodes’ behavior, employing a deep learning model (Algorithm 2) for malicious node detection. Secure nodes are connected to the RSU, forming clusters, and each RSU within a cluster assigns authentication keys to cluster members. The RSU is responsible for optimizing communication paths, detecting and handling malicious nodes, and ensuring secure clusters through key assignments. The RSU, through Algorithms 1 and 2, orchestrates the entire process. It evaluates vehicle speed, direction, and neighboring nodes, forming clusters and facilitating secure connections. The RSU efficiently utilizes a deep learning model to detect and exclude malicious nodes, ensuring the integrity of the clusters. It manages content distribution and facilitates message requests and responses within the formed clusters. The RSU’s continuous monitoring of vehicular nodes and dynamic adjustments to connectivity parameters contribute to a robust and adaptive communication framework. This comprehensive approach ensures efficient content delivery, robust cluster formation, and resilient communication architecture, which is crucial for the proposed system’s success in vehicular networks.
**Algorithm 1.** Cluster formation**Input:** 
$V_{cl}^{vel}$

$,\;V_{cl}^{loc[x,\;y]}$

$,\;\mathrm{map}\_\mathrm{lans},\;\mathrm{tim}\_\mathrm{span},\;{RSU}_{i}$

$,\;N_{neighbour}$
**Output:** Cluster, Number of Clusters **Steps:**
  1.
While t← 1 to tim_span  2.
   While i ← 1 to map_lans  3.
$\;\;\;\;\;\mathrm{if}\;(\mathrm{detect}(\;{RSU}_{i}$ ))  4.
$\;\;\;D_{RSU}^{Node}\leftarrow$ $\left(\;V_{cl}^{loc[x,\;y]},\;{RSU}_{cl}^{loc\left[x,\;y\right]},\;N_{Neighbour}^{Node}\right)$ :  5.
$\;V_{cl}^{vel}\;\;\;\;\leftarrow$ vehicle speed  6.
$\;if((D<D_{threshold})\;||\;(V_{cl}^{vel}<V_{cl}^{vel(threshold)})||\;(N_{Neighbour}^{Node}<N_{Neighbour}^{threshold})\;)||$ ( $V_{N}\;\;\;$ ← Algorithm 2))   7.
$\;\;{RSU}_{i}\;\leftarrow \;\;ID$
  8.        End if  9.
$\;\;\;C_{i}$ ← ${RSU}_{i}\left(\forall ID\right)$
  10.   End if  11.     End  12. End  13.
$\;\mathrm{set}(C_{i}^{Range})$
  14.
$\;Set(C_{i}^{Identity}$ )  15.
$\;C_{i}^{N}\;\leftarrow \;\forall C_{i}\;$ $∴\;where\;C_{i}\;\neq \;0$



This continual process involves connecting and influencing vehicles and ensures a steady data flow and an efficient, reliable communication framework. It is important to note that we assume all vehicles move in the same direction. In a real-world context, variations in speed would be observed, but our approach accommodates these fluctuations by considering the relative speed, thus maintaining an efficient and reliable communication architecture.

Furthermore, the speed of each vehicular node is monitored to enhance content delivery efficiency. If a vehicle’s speed falls below a certain $V_{cl}^{val}$ , the threshold is permanently associated with the RSU. After verifying the connectivity parameters within the cluster, our system identifies and flags malicious nodes that exhibit suspicious behavior. The selection of malicious nodes is achieved through a deep learning process, significantly enhancing the efficiency of learning and detection, as detailed in Algorithm 2. We employ logistic regression, a binary classification algorithm, and ANN, a supervised deep learning technique, to identify black holes or malicious nodes. A precise and accurate model is cultivated using backpropagation, a method for minimizing error by reducing the cost function. Nodes identified as abnormal or malicious are subsequently excluded or rejected, while secure or normal nodes are integrated into the cluster. A three-layered deep learning model is deployed to detect and classify malicious attacks within the proposed system. Artificial neurons and successive layers process the input data, ultimately evaluating architectural values to pinpoint black holes or malicious or counterfeit nodes. Training the deep learning network hinges on detecting malicious attacks based on parameters such as black hole attack intensity, packet drop ratio, data content, timestamps, packet rates, and the distance between nodes and RSU in the network. This architectural approach repetitively uses 25, 50, 75, and 100 nodes to detect malicious nodes with black hole attacks. Once malicious nodes are identified, communication paths are determined using a modified AODV-based routing table and updated following the provided AODV architecture, as expounded in Algorithm 2, detailing the deep learning model for malicious node detection.
 **Algorithm 2.** Black hole attack detection through deep learning **Input:** 
$P\;as\;properties\;of\;vehicel,\;N_{type}\;for\;algorithm,\;N_{k}\;for\;Neural\;network\;$
     

$kernal\;function\;,\;{ML}_{Ilteration,}\;{ML}_{Neurons}^{Quantity},\;data-Division\left(25,50,75,100\right),\;$
 

$C_{N}^{VN}\;is\;current\;Node\;from\;all\;VNs,\;$
 
 **Output:** 
Blackhole detection and transformation
 **STEPS:** **TRAINING MODEL OF MACHINE LEARNING** 
$P_{initial}\;\leftarrow Algorithm\;2$

${kernal}_{Routing}^{ML}\left(\right)\leftarrow (initialize)$
  While k  ← 1 to ${All}_{VN}$

    if ( ${VN}_{i}\;\;\to \;{property}_{real}$ )        
${Allocate}_{VN}$ ← training_data()
     
${Train}_{1}^{to\;N}\leftarrow (VN(i))$

  
End While 
  
${ML}_{Trained}^{System}\;\leftarrow System\_Train\left(P,\;{Allocate}_{VN},\;N_{K}\right)$
  
${Trained}_{dataset}\;\leftarrow \;{ML}_{Trained}^{System}\;.\;Machines()$ **INITIALIZE ML PARAMETERS**  
for i=1 to P
     
$if\;\left(p\;\in \;{Real_{node}}_{Perty}\right)$

      
${Group1}_{real}^{nodes}()$ ← All 
   
$else\;if\;\left(p\;\in \;{Non-Real_{node}}_{Perty}\right)$
      
${Group2}_{non-real}^{nodes}()$ ← All
 
do

$\;\;\;\;\;\;\;{Group3}_{extra}^{nodes}()$ ← (extra)
 END 
${Network}_{Trained}\;\leftarrow NewoffTrained(VN,\;G1,\;G2,\;G3,\;P)$
**STING** 
$While\;j=1\;to\;{All}_{Trained\;Nodes}$
        
$Process\left(CN\right)\;\leftarrow Current\;Node\;Properties\left(\right)\;\;$
             
∴get current node priperties
       
$R_{Auth}\;\leftarrow simulation(C_{N}^{VN},\;$ ${Network}_{Trained})$
       
$if(R_{Auth}=TRUE)$
          
${Auth}_{Node}\;\;\;\leftarrow TRUE$ Authenticated Node Exists
       
else 
          
${Auth}_{Node}\;\;\;\leftarrow FALSE$
 END 
$Return:\;{Auth}_{Node}\;as\;output$



In this approach, the RSU is pivotal in guiding the formation of clusters, as detailed in Algorithm 1. Initially, each vehicle node enters the simulation environment, represented as a lane on the highway, and broadcasts a timestamp. These vehicles initially establish communication with all RSUs within their operational range. To achieve robust and stable cluster formation, we employ four critical conditions. These conditions consider the relative change in speed (denoted as $V_{cl}^{val}$ ), the distance (D) covered by the vehicle during the timestamp, and the number of neighboring vehicles within the range of cluster nodes.

The distance between an RSU and a new vehicular node is computed upon entering the system. According to Algorithm 1, the vehicular node is affiliated with the RSU if this distance is below a certain threshold. This continual process involves connecting and influencing vehicles and ensures a steady data flow and an efficient, reliable communication framework. It is important to note that we assume all vehicles move in the same direction. In a real-world context, variations in speed would be observed, but our approach accommodates these fluctuations by considering the relative speed, thus maintaining an efficient and reliable communication architecture.

Furthermore, the speed of each vehicular node is monitored to enhance content delivery efficiency. If a vehicle’s speed falls below a certain $V_{cl}^{vel}$ , the threshold is permanently associated with the RSU. After verifying the connectivity parameters within the cluster, our system identifies and flags malicious nodes that exhibit suspicious behavior. The selection of malicious nodes is achieved through a deep learning process, significantly enhancing the efficiency of learning and detection, as detailed in Algorithm 2. We employ logistic regression, a binary classification algorithm, and ANN, a supervised deep learning technique, to identify black holes or malicious nodes. A precise and accurate model is cultivated using backpropagation, a method for minimizing error by reducing the cost function. Nodes identified as abnormal or malicious are subsequently excluded or rejected, while secure or normal nodes are integrated into the cluster.

A three-layered deep learning model is deployed to detect and classify malicious attacks within the proposed system. Artificial neurons and successive layers process the input data, ultimately evaluating architectural values to pinpoint black holes or malicious or counterfeit nodes. Training the deep learning network hinges on detecting malicious attacks based on parameters such as black hole attack intensity, packet drop ratio, data content, timestamps, packet rates, and the distance between nodes and RSU in the network. This architectural approach repetitively uses 25, 50, 75, and 100 nodes to detect malicious nodes with black hole attacks. Once malicious nodes are identified, communication paths are determined using a modified AODV-based routing table and updated following the provided AODV architecture, as expounded in Algorithm 2, detailing the deep learning model for malicious node detection.

After malicious node detection, secure nodes are connected to their relevant RSU, forming a cluster. The range for every cluster is set as a fixed range with the car’s identity to be observed for the said range inside the cluster transformation. The set range is marked red and compared for the final selection of the clusters. Finally, the RSU successfully stores the ID of every vehicle with ID and becomes a thriving and active cluster member. The main ideas of Algorithms 1 and 2 are provided here.

Algorithm 1 outlines the steps for cluster selection and appointing a cluster head in vehicular networks. It iteratively assesses vehicles and RSUs, evaluating distance, speed, and neighboring nodes. When detecting an RSU, it calculates the distance between the RSU and the vehicular node, considering speed and neighbouring nodes. If conditions regarding distance, speed, and neighbors are met, the RSU is assigned an identity, and a cluster is formed. The algorithm then sets the range and identity for the cluster, ensuring its integrity. The resulting cluster, with non-zero identifiers, represents a cohesive group in the vehicular network, ready for subsequent operations like content distribution and secure communication.

- Initially, a vehicular node broadcasts a timestamp (ML parameter) to RSU. Then, RSU checks the speed of node i-e. If it is less than the speed threshold, it is considered eligible for permanent connection with RSU; if it is greater than the threshold, it forms a temporary connection with RSU, then the direction of the node is checked.
- After that, all the ML parameters are applied for malicious node detection. The node’s packet delivery and packet drop information, i.e., the node’s previous behavior, is taken from the previous RSU.
- After verifying present and previous behavior through the deep learning classifier ANN, the node is declared secure and becomes part of the secure cluster.

Ultimately, each RSU within a cluster assigns an authentication key to every member of the secure cluster. The parameters for cluster formation are $V_{cl}^{vel}$ the velocity of vehicles reached in the cluster domain, $V_{cl}^{loc[x,y]}$ shows the location of vehicles for the cluster erra transformation, map_lans shows the actual maps location of vehicles, tim_span shows the time tables from the entry into cluster and approval from the ML model, ${RSU}_{i}$ all the RSUs used in the clusters, and $N_{neighbour}$ shows the parameter for the number of neighbours of the vehicles used in the cluster formation. The aspects involved in this algorithm show the vehicle’s final selection and clear understanding. The algorithm outputs the customer and its members with the number of clusters made through the proposed approach.

The complexity of Algorithm 1 for cluster formation involves nested loops iterating over time spans and map locations, making it dependent on the size of the input space (tim_span and map_lans). Within these loops, the algorithm performs conditional checks and calculations based on parameters such as vehicle speed, distance thresholds, and the number of neighbors. The number of RSUs, the conditions for cluster formation, and the execution of Algorithm 2 influence the overall time complexity. The complexity is likely polynomial, significantly impacting the nested loops and conditional statements.

Input parameters are the key parameters based on which the algorithm selection process works. $N_{\mathrm{type}}$ is used for the type of ML algorithm used, $N_{k}$ is the neural network kernel function, ${\mathrm{ML}}_{\mathrm{Alteration},}$ shows the number of iterations of an ML model speech, ${\mathrm{ML}}_{\mathrm{Neurons}}^{\mathrm{Quantity}}$ shows the quality of NN neurons in the ML model, $\mathrm{data}-\mathrm{Division}\left(25,50,75,100\right)$ shows the clear division of data into sections, and $C_{N}^{\mathrm{VN}}$ shows the selected nodes from all cluster nodes. The output parameter is the final selection vehicle that is malicious or non-malicious.

The training and testing phases of the machine learning model determine the complexity of Algorithm 2. The training involves iterating over the nodes in the network (All_VN), with nested loops for property allocation, model training, and parameter initialization. While evaluating the trained model, the testing phase involves iterating over the trained nodes and simulating the authentication process. The time complexity is influenced by the number of nodes, neurons in the neural network, and the iterations involved in training. Additionally, the use of deep learning techniques suggests a complexity that is likely high and depends on the complexity of the neural network architecture.

### 3.5. Path Prediction

Path projection is achieved by implementing an enhanced AODV. In the standard AODV protocol, three operational modes exist:

- **Route Request Mode:** In this mode, a request for a route is disseminated to all neighboring nodes, persisting until it ultimately arrives at the intended destination.
- **Route Reply Mode:** In this mode, each neighboring node that can potentially offer a route to the destination responds by providing the route information to the source node. The source node then forwards the message via the route characterized by the most prominent sequence number.
- **Route Maintenance Phase:** This phase comes into play when a route fails to transmit a message to the destination.

#### 3.5.1. Route Request and Route Reply

In the initial phase, the source node initiates the transmission of a forward communication path (FCP), including a timer, to the neighboring nodes. This path traverses through all nodes until it successfully reaches the destination node. Moving from one node to another, it accumulates and stores data concerning the entire set of visited nodes. These data encompass details such as the identification of the visited nodes, the time of arrival at each node, the distance of each node from the source, and the node’s capacity, denoted by the number of channels it possesses. Upon reaching the destination node, the system generates a reverse communication path (RBP), and the FCP is subsequently dismantled. Following the deactivation of the FCP, the destination node crafts a bidirectional communication path (BCP) and dispatches it back to the source node using the same route. During this process, the system computes the distance between the source and destination nodes and the number of intermediary nodes along this route. The source node receives the RBP from multiple neighboring nodes, while the BCP offers multiple routes to the source node for transmitting packets. The modification in the creation and management of the FCP involves the transmission of a forward communication path (FCP) from the source node to the destination node. This path accumulates and stores data regarding visited nodes, arrival times, distances from the source, and node capacity. The FCP is deactivated upon reaching the destination, generating a bidirectional communication path (BCP), which is sent back to the source using the same route. The BCP provides multiple routes for transmitting packets, and Equation (1) is employed to calculate distances along these routes. The introduced parameters include node identification, arrival times, distances, and node capacity, contributing to a more comprehensive and detailed path establishment than the standard AODV. However, it introduces a forward communication path (FCP) and reverse communication path (RBP) in the initial phase, along with a bidirectional communication path (BCP) during the route reply process. The details suggest an enhanced mechanism for path establishment and data accumulation compared to the traditional AODV. Equation (1) is employed to calculate the distances along these routes.

(1) $${Dis}_{N}=\frac{{Dis}^{2}\left(S,D\right)+{Dis}^{2}\left(S,n\right)-{Dis}^{2}\left(n,D\right)}{2\times {Dis}^{2}\left(S,D\right)}.$$

The introduced forwarding candidate path (FCP), repairing backup path (RBP), and backup candidate path (BCP) in the modified AODV protocol significantly contribute to improved efficiency, reduced latency, and enhanced reliability. FCP strategically identifies and utilizes optimal paths for data forwarding, minimizing congestion and enhancing overall network efficiency. RBP acts as a proactive measure, swiftly repairing broken links to maintain seamless communication, thereby reducing latency. BCP adds a layer of reliability by establishing alternative paths in advance, mitigating the impact of link failures and ensuring robust connectivity. Collectively, these enhancements result in a protocol that not only optimizes resource utilization but also minimizes data transfer delays and fortifies the network against potential disruptions, thereby elevating the overall performance and dependability of the AODV protocol.

#### 3.5.2. Route Maintenance

Upon route establishment, the system selects the optimal pathway from the routing table, aiming for the one that offers the shortest distance and the least number of hops. The path selection criteria with minimal transmission delay may shift depending on the scenario. An objective function is introduced, driven by decision variables, to facilitate the achievement of this objective. These decision variables encompass hop counts, distance, and node capacity, aiming to optimize packet transmission delay. The delay experienced in transmitting packets is contingent upon these decision variables. Following a comprehensive, objective function evaluation, the source node dispatches the packet to the destination. If path errors are detected, the roadside unit (RSU) broadcasts a route error message back to the source. Subsequently, the source node redirects the packet through an alternative path that is the next most suitable. It is important to note that this communication method applies exclusively when the destination is located within the cluster. However, if the destination node is situated outside the cluster and requires communication with subsequent vehicular nodes, a mechanism comes into play. When the destination is within the cluster, the communication process involves RSU broadcasting a route error message back to the source node upon detecting path errors.

The source node then redirects the packet through an alternative path within the same cluster. However, an authentication key mechanism is activated if the destination is outside the cluster and requires communication with subsequent vehicular nodes. The RSU furnishes an authentication key in Algorithm 1, and upon RSU confirmation, the vehicular node can communicate with the next cluster node, mirroring the process for inter-cluster communication. The RSU authentication key serves a crucial role in inter-cluster communication. When a vehicular node needs to communicate with subsequent nodes outside its cluster, it utilizes the authentication key provided by the RSU in Algorithm 1. This key serves as a form of authorization, allowing the vehicular node to establish communication with the next cluster node. The RSU’s authentication key acts as a secure credential, ensuring that only authorized nodes within the cluster can communicate with nodes in other clusters. This mechanism enhances the security and integrity of inter-cluster communication by controlling access and verifying the legitimacy of communication between clusters in the vehicular network.

In this scenario, the authentication key furnished by the RSU in Algorithm 1 is shared with the following cluster node. Following RSU confirmation, the vehicular node can communicate with the next cluster node. The process mirrors the one employed for inter-cluster communication.

Various procedures and loops influence the complexity of Algorithm 3 for path prediction using modified AODV. The FCPdata_send procedure involves sending messages to neighbors, and the objective function procedure utilizes AODV to obtain paths and evaluate fitness functions. The overall time complexity is likely influenced by the number of vehicular nodes, the iterations in evaluating fitness functions, and the conditional checks for distance, hop count, and channel availability. The nested loops contribute to the complexity, especially in the objective function procedure. The overall complexity is likely polynomial and depends on factors such as the number of nodes, paths, and the effectiveness of the fitness function evaluation.
**Algorithm 3.** Path prediction using modified AODV**Input**
: secure clusters, FCP,BCP

$,\;vehicular\;nodes\left(25,50,75,100\right)$
**Output:** Predicted path, targeted path
 
FCPdata_send(source, destination)
   
if(neighbor!=0)
       Send FCP to all neighbors     
for i=1 to n { 
if(neighbor=destination) { {    Delete FCP and create BCP   
Data_send(source, destination) }Else   
FCPdata_send(source, destination) }   Procedure Objective function()   
Available paths →   AODV
 While (j<=p) Obtain path and evaluate fitness function      
if(distance, hopcount and channels>threshold)

   Evalaute  obejctive function()Increment jEnd whileEnd


## 4. Performance Evaluation

This chapter delineates the context and enacts the envisioned project within the VANET milieu. Herein, we elucidate the outcomes of our experiments, employing deep learning techniques and simulations conducted using the Network Simulator-2 (NS-2). These endeavours are aimed at comprehensively assessing and executing the principal operational scenarios.

### 4.1. Simulation Scenario

We implemented the prescribed methodology in NS-2.33 to assess the efficacy of our proposed model. This model was meticulously crafted to ensure vehicular devices’ efficient and dependable performance in routing and detecting black hole attacks. The critical parameters for gauging the system’s performance encompass end-to-end delay, network throughput, average hop count/throughput, and average delay. We assessed these parameters by comparing results referenced in the literature [4,36]. Our comprehensive evaluation unfolds within the expansive confines of the NS-2 simulator, covering 1400 m by 1400 m. The experimental parameters and configurations are meticulously detailed in Table 2. To appraise the performance of our proposed protocol, we scrutinized a custom network topology replete with interaction points, vehicles, highways, RSUs, source vehicles, and destination vehicles. The efficiency of the revised-AODV protocol was assessed based on crucial parameters, including end-to-end delay, network throughput, average hop count, and average delay. These metrics serve as quantifiable indicators, offering a clear basis for comparing AODV approaches. By focusing on these specific factors, the paper establishes a comprehensive framework for evaluation, ensuring a legitimate and justified comparison between the proposed algorithm and existing AODV variations.

Furthermore, in our evaluation, we incorporated node mobility and the count of active connections within the network as pivotal parameters for the ultimate test of identifying malicious nodes. Our study’s vehicles traversed roadways while communicating with the roadside infrastructure. This communication transpired via Wi-Fi connectivity, enabling vehicles to interact with one another and with RSUs. Such interactions are essential for content requests and subsequent content delivery, governed by specific parameters.

Vehicles traveling in various directions and along different routes also established connections with the next RSU and other vehicles within their cluster range. Despite adopting cluster-based routing, each cluster adhered to its unique routing protocols. When a new vehicle entered the range of a cluster and became a member, it solely solicited content from the designated RSU. We employed 100 vehicles in our simulation, randomly distributing their internal distances within a given cluster. The road width was standardized at 3 m, with all roadside elements remaining stationary. This focus on V2V communication rendered the vehicles immobile and exclusively operated within urban roadside areas. The average speeds of these vehicles fluctuated within predefined limits, and the simulation duration, as indicated in Table 2, was extended to surmount challenges, thereby facilitating vehicle travel. We gauged the performance of our model through a set of key performance indicators (KPIs). These included an ANN-based intelligent network model for black hole detection, measuring the training and testing accuracy to minimize error rates in detecting black hole attacks. We also quantified hop count to ascertain the minimum hops in communication paths from source to destination, gauged end-to-end delay by calculating the ratio of packets sent and received at the destination node, and measured network throughput.

### 4.2. Dataset and Evaluation Results

We employed a supervised deep learning model to identify malicious nodes. This model was meticulously trained using pre-existing datasets, as documented in reference [36], which pertained to detecting malicious nodes within wireless sensor networks. Our dataset encompassed 14 independent variables and a singular dependent variable. The independent variable attributes included data such as destination IP, next hop, hop count, vehicle information, RSU’s, ANN algorithm, parameters, malicious node, RSU-based decision, route request, route reply, sequence number, classification, packets delivered, and shortest path (yes/no). In this case, the output variable pertained to the class or category of the nodes and the shortest path for the variable values. Our dataset was substantial, consisting of more than 50,000 records. We employed an ANN classifier to detect malicious nodes. Table 3 provides the layers with output parameters and values from each NN layer in the system.

**Figure 9:**
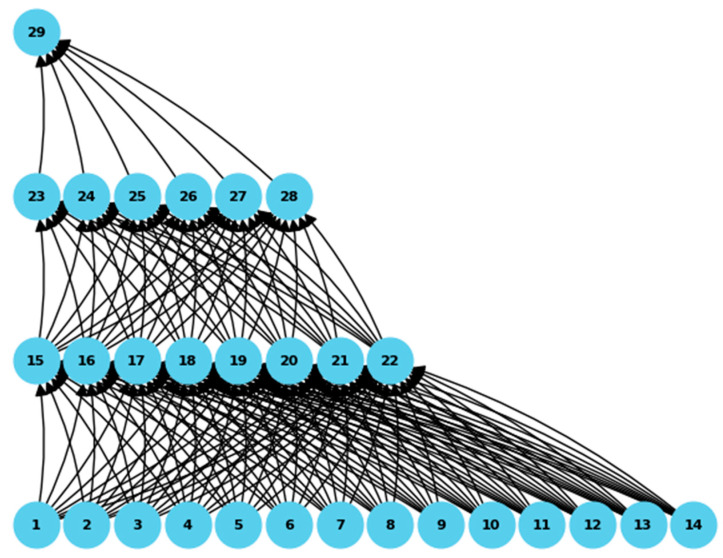
Input layer, hidden layer, and output layer of the NN model.

Figure 9 shows NN, input, hidden, and output layers. In the context of network routing and decision-making, the configuration of a neural network plays a pivotal role in optimizing performance. A neural network designed for this purpose may comprise, for instance, 14 input nodes representing relevant network parameters, two hidden layers containing 8 and 6 nodes, respectively, and a single output node for decision output. This architecture enables the model to capture intricate patterns and relationships within the data, facilitating complex decision-making processes. The choice of layers and nodes is informed by the specific requirements of the routing task to balance model complexity and computational efficiency.

Moreover, leveraging activation functions such as the rectified linear unit (ReLU) and employing dropout techniques at 0.2 between layers enhances the network’s ability to generalize and prevent overfitting. Such a well-configured neural network demonstrates the capacity to learn and adapt to dynamic network conditions, ultimately contributing to efficient and adaptive routing and decision-making in complex networking environments. Our approach adopted gradient-boosted neural networks (GBNN) as an ANN model. This hybrid approach combines the strengths of gradient boosting and neural networks. It trains a neural network to capture complex patterns in the data and integrates it with a gradient-boosting framework. GBNN is effective for improving predictive performance on structured tabular data. The gradient-boosted neural network (GBNN) model presents a compelling fusion of the strengths derived from gradient boosting and neural networks, offering a unique set of advantages compared to standalone models. One notable advantage is the model’s ability to capture intricate patterns within structured tabular data. By training a neural network within a gradient-boosting framework, GBNNs can effectively learn complex, non-linear relationships that may exist among different features. This is particularly advantageous in scenarios where traditional linear models may fall short, as the neural network component allows for extracting high-level abstractions and representations from the data. Another significant advantage lies in the ensemble nature of GBNNs. Integrating gradient boosting with neural networks results in a hybrid model that leverages the strengths of both approaches. The boosting framework helps to improve the model’s overall predictive performance by sequentially correcting the errors made by the neural network component. This collaborative learning process often leads to enhanced generalization capabilities, making GBNNs well-suited for a wide range of tabular datasets.

We gauged the performance of this classifier using the BHT dataset, as documented in reference [39]. This evaluation was predicated on the forecasted outcome values, and Table 4 comprehensively depicts this anticipated dataset’s overall performance. An expanded array of features bolstered the system’s performance metrics. Accuracy, as a performance metric, was calculated by determining the ratio of correct predictions the model made to the total number of predictions. Equation (2) outlines the computational formula employed for the accuracy assessment of the GBNN model. The accuracy precision and recall values were computed using parameters such as true positive (TP), true negative (TN), false positive (FP), and false negative (FN).

(2) $$Accuracy=\frac{TP+TN}{TP+TN+FP+FN}.$$

Precision was computed by ascertaining the number of accurate positive predictions made by the model and dividing this by the total count of positive predictions. Equation (3) delineates the formula used for computing precision in the context of the ANN model.

(3) $$Precision=\frac{True\;Positives}{True\;Positives+False\;Positives}.$$

Recall was determined by quantifying the number of correct positive predictions generated by the model and dividing this figure by the total count of positive instances within the dataset. Equation (4) elucidates the mathematical formula utilized for calculating recall within the framework of the ANN model.

(4) $$F1-Score=\frac{\left(TP\right)}{\left(TP+FN\right)}.$$

The F1 score is a metric that assesses the equilibrium between precision and recall. This metric was computed as the harmonic mean of both precision and recall, as demonstrated by Equation (5), which provided the formula for calculating the F1 score within the context of the ANN model.

(5) $$Precision=\frac{2\times (Precision\times Recall)}{\left(Precision+Recall\right)}.$$

### 4.3. Model Accuracy

**Figure 10:**
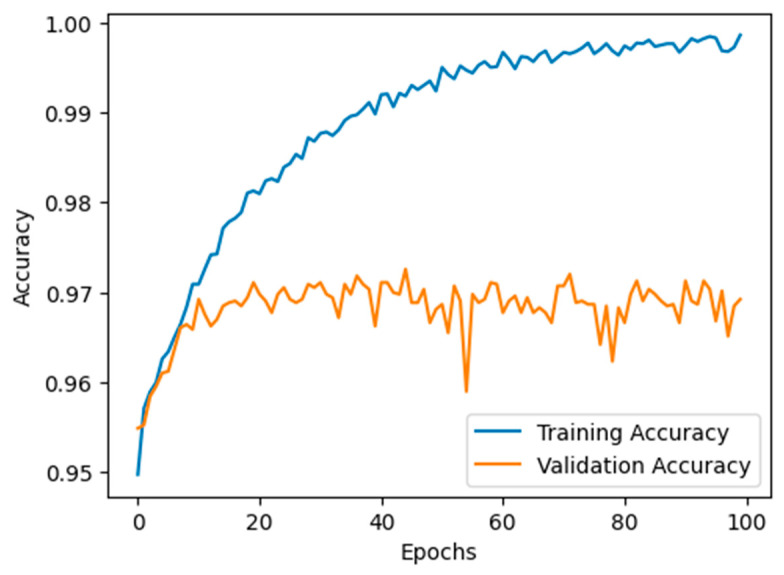
Model accuracy of the proposed BHT model.

Building upon the performance evaluation of the proposed system concerning the BHT dataset, we compiled a comprehensive set of outcomes to facilitate the effective analysis and prediction of system performance metrics. Our training efforts demonstrate that the training prediction of the system has undergone significant enhancements, enabling valuable performance comparisons across various datasets. Figure 10 vividly presents the model’s accuracy, further detailed in Table 4. Model accuracy, as assessed using Equation (2), offers insights into the system’s performance.

**Figure 11:**
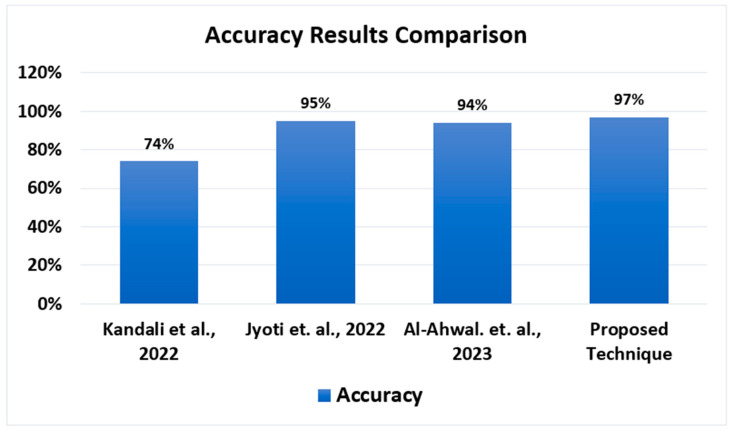
Model accuracy graph comparison with other techniques [40,41,42].

In addition to the system’s precision, the model demonstrates a noteworthy reduction in the system metric, signifying remarkable accuracy levels. The epoch values that underpin the model’s accuracy and the subsequent improved results are of particular significance for research and analytical purposes. A meticulous validation test was conducted using the carefully selected BHT model dataset, encompassing 100 epochs, to yield the model’s results. The graphical depiction, with blue lines tracing the model’s loss during the training and its testing accuracies, provides valuable insights. The proposed ANN model was methodically trained to assess its performance meticulously. The training phase was conducted on 70% of the dataset, while the testing phase encompassed 30% across all classification categories. The training and testing model results were quantified using Equations (2)–(4). The percentages denoting accuracy, precision, recall, and F1 score, as displayed in Table 3, underscore the elevated accuracy levels that persistently showcase the model’s efficacy and the functions that govern its performance. Figure 11 shows the model accuracy comparison with other techniques.

### 4.4. Model Loss

**Figure 12:**
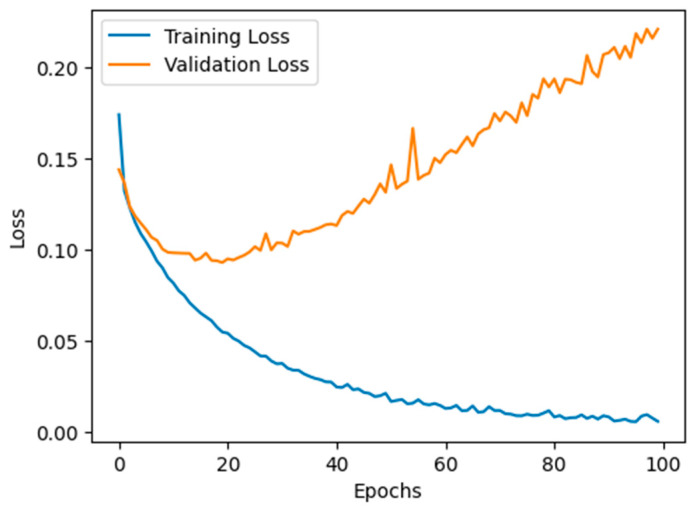
Model loss for the BHT dataset.

Figure 12 visually portrays the model’s loss concerning the proposed BHT dataset, and the associated accuracy results are briefly elaborated in Table 3. This graphic presentation encapsulates the loss experienced by the suggested system across the designated dataset. System performance evaluation hinges on utilizing training and test datasets, focusing on the model’s loss metrics. These metrics align with the predefined dataset, thus enabling the iterative training and testing of the proposed model. The graphical representation of the model’s loss is visually depicted in Figure 12.

In this configuration, the vehicles within the system employed UDP to transmit data packets, with the transmission rate varying from 0.5 Mbps to 5 Mbps. In contrast, each packet was approximately 512 bytes in size. The key parameters assessed for performance evaluation encompassed end-to-end delay, packet delay, and network throughput. Additionally, we carefully monitored the occurrences of packet drops and inter-vehicle communication. Each simulation scenario was rigorously executed 150 times to ensure comprehensive and reliable results. Table 5 shows the prediction performance of the BHT model with vehicle speed in m/s and simulation time in the simulation environment. The table content shows the number of vehicles with speed under different simulation times to capture the malicious nodes effectively. Implementing ML is a pivotal tool to enhance routing efficiency. ML primarily manages traffic flow, endeavors to minimize packet transmission delays, and enhances the system’s ability to detect malicious attacks. In black hole attacks, there is a discernible impact on the packet transmission ratio, resulting in increased hop counts for network transmission and ensuing delays. By introducing machine learning into the routing process, we sought to augment the overall performance and refine the criteria for detecting and mitigating malicious attacks within the network.

### 4.5. End-to-End Delay

**Figure 13:**
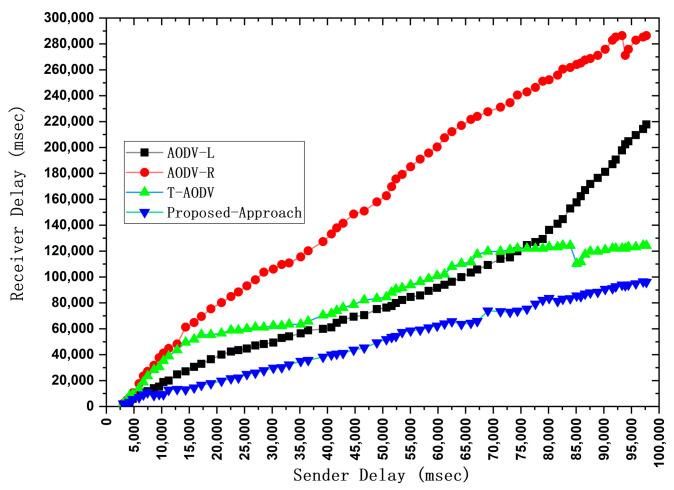
End-to-end delay for packet delivery.

In the context of vehicular ad hoc networks (VANET), end-to-end delay pertains explicitly to measuring the time it takes for a packet to be transmitted from the source node and successfully received at the destination node during the black hole attack scenario. The behavior of a black hole attack depends on the routing procedure, protocols, and the number of involved nodes. The results in Figure 13 compared existing techniques with the minimum number of times the under-attack scenario was observed. The less time shown here effectively affects the network routing performance less than existing techniques. This metric is paramount during routing, reflecting data transfer efficiency in the VANET environment. This dynamic capability of nodes to send and receive packets directly influences the end-to-end delay, representing the culmination of packet delivery and transmission ratios. We maintained detailed records concerning the specific setup characteristics and network assumptions, which are pivotal in estimating end-to-end delays. The observed delay was a valuable indicator of various aspects, including the spatial positioning of neighboring vehicles and any routing alterations caused by malicious nodes. Furthermore, the count of hops required for data transmission between the sender and receiver nodes provided insights into the effectiveness of data transmission, especially given the evolving nature of the network. In Figure 13, we graphically illustrate the end-to-end delay in the network, comparing the results achieved by our proposed technique with those of existing approaches.

**Figure 14:**
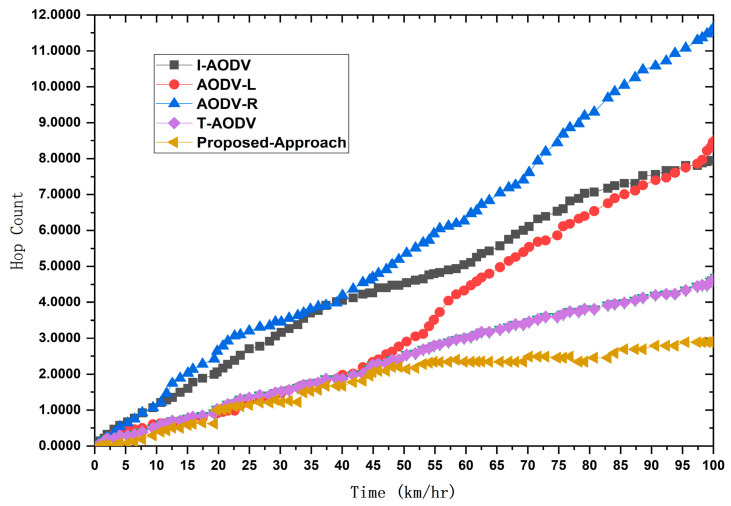
Average hop count for proposed methodology in the network.

The proposed methodology demonstrates an impressive reduction in end-to-end delay within the network, marked by minimal latency in the transmission of data packets between senders and receivers, quantified in milliseconds. As showcased in Figure 14, this reduction was notably pronounced in scenarios with varying vehicle densities and speeds, where the efficiency of transmitting and receiving packets between sender and receiver vehicle nodes is paramount. Comparatively, other techniques exhibited considerably higher delays within the network, affecting the efficiency and reliability of data delivery. In contrast, the proposed approach consistently performed superiorly, showcasing minimal delays across the network’s diverse scenarios.

### 4.6. Average Hop Count

**Figure 15:**
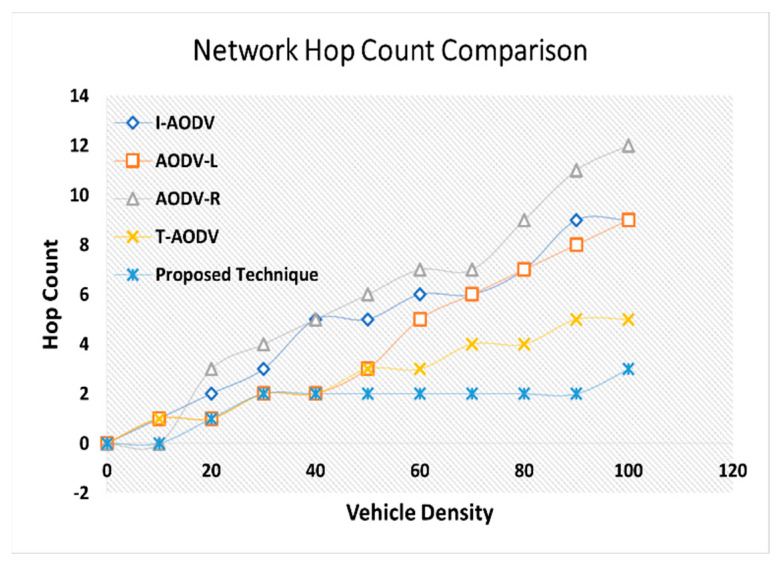
Hop count comparison with proposed technique.

**Figure 16:**
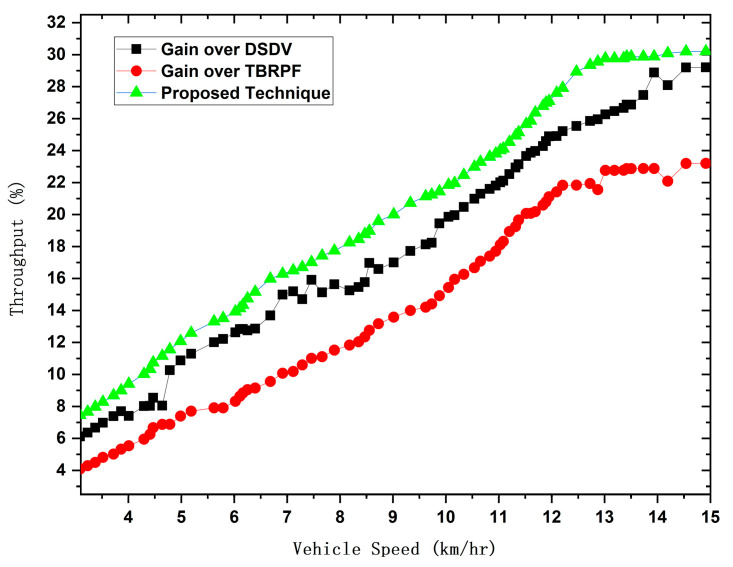
Network throughput for proposed technique as compared with existing results.

Average hop count (AHC) is a pivotal network simulation parameter within the VANET context. This metric rationalizes the trajectory of network packets as they traverse from consumer nodes to producer nodes reciprocally. The AHC meticulously tracks the number of neighboring nodes passing by these packets, utilizing the designated simulation parameters. The results rendered by the AHC yield predictive insights into the number of hops required for data transmission from producer nodes to consumer nodes within VANET. Figure 14 vividly illustrates these results, offering a comparative analysis with established network techniques. Furthermore, Figure 15 presents a comprehensive comparison of hop counts with well-known routing strategies such as improved ad hoc on-demand distance vector routing (I-AODV), ad hoc on-demand distance vector routing with load balancing (AODV-L), ad hoc on-demand distance vector routing with reliability (AODV-R), and trust-based ad hoc on-demand distance vector routing (T-AODV). Results in the proposed technique were compared with those mentioned in Figure 15 to determine the average hop count and in Figure 16 for vehicular density values.

### 4.7. Average Hop Count/Throughout

Within this network, throughput was meticulously assessed following the initial positioning of vehicles and their interaction with the identified intensity levels corresponding to vehicle speeds. Figure 16 provides a graphical representation of this relationship under the black hole attack scenario. Notably, calculating packet drops was essential to this evaluation, tracking the transmission paths in various vehicle communication scenarios. Throughput essentially embodies the synchronization of comprehensive sets of data packets, encompassing their transmission and reception at diverse speeds to evaluate network performance. A comparative analysis of throughput was performed, pitting the proposed technique against well-established protocols, including the destination-sequenced distance-vector routing protocol (DSDV) and the temporally ordered routing algorithm for mobility (TORA) based on the reverse-path forwarding protocol (TBRFP). The outcomes underscore the proposed technique’s superiority in network throughput, underscoring its efficiency in routing for enhanced network performance under a black hole attack scenario.

### 4.8. Average Delay

**Figure 17:**
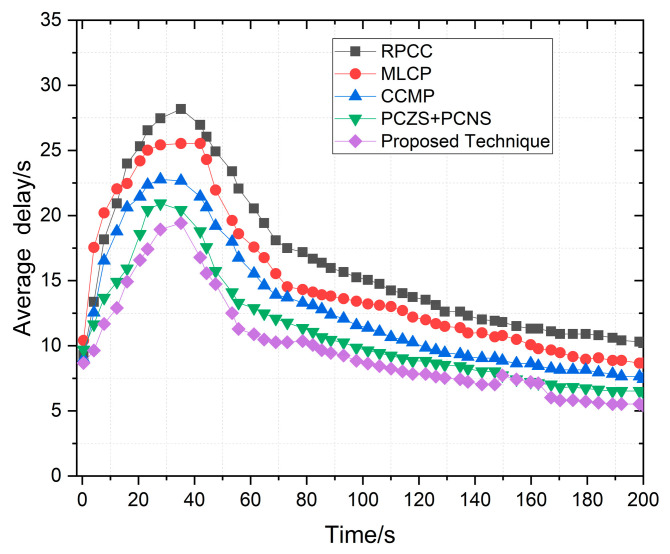
The average delay of the proposed technique was compared to MLCP, RPSS, CCMP, and PCZS + PCNS [1].

The “Average delay” refers to the time between processing data requests from different networks or zones. We carefully evaluated this “Average delay” using simulations with between 50 and 150 trucks. The basis for assessing the results of our suggested system was the following two crucial factors. Figure 17 provides a concise graphical depiction of the results, making it easier to understand the outcomes that our creative method produced. The trends that were observed point to an early rise in the average delay, which is followed by a leveling out of the delay and, finally, a thorough summary of the outcomes. These outcomes clarify the overall approach and the definitive conclusions, which support the effective management of the system. While the material cached in various zones was nonexistent at the beginning of the simulation, it eventually led to the effective use of our suggested methods. We eventually contrasted the findings obtained from our recommended method with those obtained from improved coverage, and connectivity in heterogeneous wireless sensor networks was improved. Routing protocol for coverage and connectivity (RPCC) was proposed in which point coverage and connectivity was enhanced. MLCP maintained a hierarchical topology, where elected cluster-heads at the lower level did not participate in the next cluster-head election. Each member of the high-level cluster came from one lower-level cluster [43]. On-demand routing protocol DSR with a CCMP-AES mode to defend against black hole attacks provided confidentiality and authentication of packets in both routing and link layers of MANET [44]. Payment channel networks (PCNs) were designed and utilized to address the scalability challenge and throughput limitation of blockchains. Routing is a core problem of PCNs. An ideal PCN routing method needs to achieve (1) high scalability that can maintain low per-node memory and communication costs for large PCNs, (2) high resource utilization of payment channels, and (3) the privacy of users [45]. These outcomes significantly improve the suggested approach’s operational effectiveness. Figure 17 illustrates how network latencies of different intensities show a significant decrease, improving the effectiveness of cooperative results.

## 5. Conclusions, Limitations, and Future Directions

The proposed methodology was embraced to address issues regarding malicious nodes, which often lead to packet drops due to their security activities. Additionally, this approach encompassed the task of forecasting and identifying optimal routes, effectively employing a modified version of the AODV routing protocol. An intelligent cluster-based routing strategy was harnessed to heighten routing efficiency within the network. Previously, detecting malicious nodes rested with RSUs, which employed an ANN classifier to scrutinize nodes’ behavior and discern suspicious activities. Following the establishment of secure clusters, the modified AODV protocol takes center stage, facilitating the dynamic formation of on-demand paths for packet transmission. This intricate protocol leverages forward and backward control packets during route discovery and employs objective functions to meticulously determine the most optimal route. The results obtained from this methodology were judiciously compared with pre-existing routing techniques. The comparative analysis illustrated the efficiency of our proposed approach, which notably excelled in average hop count, end-to-end delay, and network throughput.

The main limitations of the research are as follows:

- This study was confined to identifying black hole attacks within the VANET environment.
- It is worth noting that neural networks are susceptible to adversarial attacks, which can potentially induce erroneous results within the network. This vulnerability becomes particularly pronounced in vehicular ad hoc networks, where malicious nodes can readily manipulate data.
- Detecting black hole attacks primarily centers on routing aspects but is delimited to the research dimensions concerning network load balancing and its intricacies.
- While proficient in identifying the shortest, most secure, and efficient routes from source to destination nodes, the research was limited to facilitating confidential communications exclusively among vehicles engaged in specialized convoy operations, which have implications for national security.

Using the reinforcement-learning model, our proposed technique will be refined by adapting secure V2V communication with minimum delay.

## Figures and Tables

**Table 1:** Modified AODV routing protocol and its implementation.

Destination IP	Next Hop	Hop Count	Vehicle Information	RSU’s	ANN Algorithm Parameters	Malicious Node	RSU based Decision	Route Request	Route Reply	Sequence Number	Classification	Packets Delivered	Shortest Path (Yes/No)
192.168.1.100	192.168.1.105	2	Vehicle 1 (Sedan)	RSU1, RSU3	Learning Rate: 0.01	No	Yes	4	2	1234	High	1000	Yes
192.168.1.201	192.168.1.202	1	Vehicle 2 (Truck)	RSU2, RSU4	Learning Rate: 0.02	Yes	No	3	1	5678	Medium	800	No
192.168.1.305	192.168.1.308	3	Vehicle 3 (Motorcycle)	RSU1, RSU2	Learning Rate: 0.01	No	Yes	5	3	9876	Low	1200	Yes
192.168.1.410	192.168.1.412	2	Vehicle 4 (Bus)	RSU3, RSU4	Learning Rate: 0.03	No	Yes	2	1	3456	High	900	No
192.168.1.501	192.168.1.504	1	Vehicle 5 (Sports Car)	RSU1, RSU2	Learning Rate: 0.02	No	No	6	4	6543	Medium	1500	Yes
192.168.1.610	192.168.1.611	1	Vehicle 6 (Van)	RSU3, RSU4	Learning Rate: 0.01	No	Yes	4	2	7890	Low	1100	No
192.168.1.701	192.168.1.703	3	Vehicle 7 (Electric Car)	RSU1, RSU2	Learning Rate: 0.02	Yes	No	2	2	4321	High	1700	Yes
192.168.1.810	192.168.1.815	2	Vehicle 8 (SUV)	RSU1, RSU3	Learning Rate: 0.03	No	Yes	3	3	8765	Medium	950	Yes
192.168.1.910	192.168.1.912	1	Vehicle 9 (Compact Car)	RSU2, RSU4	Learning Rate: 0.01	No	No	4	2	2345	Low	1300	No
192.168.1.1000	192.168.1.1003	3	Vehicle 10 (Tractor)	RSU1, RSU2	Learning Rate: 0.02	No	Yes	5	4	5670	High	1600	Yes

**Table 2:** Simulation setup and its range for effective routing and parameter discussion.

Parameters	Values
Simulator	SUMO 1.19 + NS-3.0
Network Area Range	2500 m × 2500 m
Vehicle Drive Time	200 s
Total Simulation Time	1000 s
Nodes Density	100, 150, 200, 250, 300, 350, 400.
Wireless Protocol	802.11 b
TransmissionRange Among Vehicles	1500 m
Number of Vehicles	200
Road Conditions	Two Way Highly Road
Number of RSUs	30
RSU Wireless Area	250 m
RSU Broadcast Time Interval	50 s
Network Connectivity	5G

**Table 3:** NN model with layer parameters and output values.

Parameters	Values
Dense (Dense)	(None, 14)
Dense_1 (Dense)	(None, 64)
Dense_2 (Dense)	(None, 64)
Dense_3 (Dense)	(None, 64)
Dense_4 (Dense)	(None, 1)

**Table 4:** ANN Model parameter results over classification classed 0, 1 and average.

Classification Class	Precision	Recall	F1 Score	Accuracy
0	0.97	0.99	0.98	0.97
1	0.98	0.98	0.98	0.98
Average	0.975	0.985	0.98	0.975

**Table 5:** Prediction performance for the BHT model.

Vehicle Speed (m/s)	Simulation End Time
15	56
14	57
13	59
12	61
11	64
10	67
9	70
8	75
7	80
6	88
5	99
4	115
3	141

## Data Availability

Data will be available on the request through proper institutional email.
